# The assessment of successful emotion regulation skills use: Development and validation of an English version of the Emotion Regulation Skills Questionnaire

**DOI:** 10.1371/journal.pone.0205095

**Published:** 2018-10-03

**Authors:** Michaela Grant, Nicholas L. Salsman, Matthias Berking

**Affiliations:** 1 Department of Clinical Psychology and Psychotherapy, University of Mainz, Mainz, Rhineland Palatinate, Germany; 2 Department of Clinical Psychology, Xavier University, Cincinnati, Ohio, United States of America; 3 Department of Clinical Psychology und Psychotherapy, University of Erlangen-Nueremberg, Erlangen, Bavaria, Germany; Brown University, UNITED STATES

## Abstract

Emotion regulation has become an important topic in mental health and psychotherapy research. Skills supposingly relevant for adaptive responses towards emotions include the abilities to be consciously aware of emotions, identify and correctly label emotions, understand what has caused and maintains one’s present emotions, modify the intensity or duration of one's emotions, accept and tolerate undesired emotions, confront situations likely to cue negative emotions, and provide effective self-support when working to cope with challenging emotions. To economically assess these abilities, a self-report measure has been developed in German and validated in various studies. To facilitate the use of the measure in English speaking countries, we have developed and validated an English version of the Emotion Regulation Skills Questionnaire (ERSQ) in a student sample (*n* = 263) and a sample of individual clinical sample (*n* = 35). Findings from this study provide significant evidence for the reliability and validity of the ERSQ. Thus, the measure can be used to assess a broad range of important emotion regulation skills in an economic way.

## Introduction

Emotion regulation has recently become a focal point in mental health and psychotherapy research [1–3] and has been increasingly incorporated into models of psychopathology [4–7]. Emotion regulation can been defined as ‘‘the extrinsic and intrinsic processes responsible for monitoring, evaluating, and modifying emotional reactions, especially their intensive and temporal features, to accomplish one’s goals” [[8]. p. 27–28]. Deficits in effective emotion regulation are assumed to contribute to the escalation and perpetuation of undesired affective states and hence to the development and maintenance of affective (and affect-related somatic) symptoms of mental disorders. Additionally, it has been hypothesized that behavioral and cognitive symptoms of mental disorders can be conceptualized as dysfunctional attempts to avoid aversive affective states even if this leads to undesired consequences in the long-term [9, 10].

Empirically, it has been shown that deficits in emotion regulation are cross-sectionally associated with symptoms of depression [11–13], anxiety disorders [14–16], borderline personality disorder [17–19], eating disorders [20–23], substance abuse [24–26], attention deficit hyperactivity disorder [27], and bipolar disorder [28]. Longitudinal studies provide evidence, that deficits in emotion regulation are not only symptoms of mental disorders but are most likely risk and maintaining factors for mental health problems such as depression [1, 29–32], anxiety disorders [33, 34], borderline personality disorder [35, 36], eating disorders [37, 38], or alcohol dependence [39].

Moreover, findings from experimental studies provide further evidence for the assumed effects of deficits in emotion regulation on psychopathology. For example, individuals formerly meeting criteria for major depressive disorder who have recovered were more likely to utilize maladaptive strategies like rumination or suppression than healthy controls [40]; individuals suffering from panic attacks tend to use more avoidant strategies when being confronted with anxiety-provoking or other types of aversive experiences than individuals without panic attacks [41]; and paranoia prone individuals prefer the use suppression over reappraisal when working to regulate their emotions in distressing social situations [42].

Finally, psychological interventions that explicitly focus on enhancing emotion regulation skills have been shown to be effective for a broad range of mental disorders. For example, emotion regulation is a core skill taught in dialectical behavior therapy [17]. Dialectical behavior therapy (DBT) has demonstrated effectiveness in reducing depression (as well as other symptoms) in individuals suffering from BPD (for review see [43] and has amassed at least preliminary evidence indicating effectiveness in the treatment of chronic depression [44, 45], substance abuse [46, 47], and eating disorders [48, 49]. Additional examples of promising treatments that focus on emotion regulation skills include treatments for PTSD related to childhood abuse [50], and veterans [51], GAD [52], eating disorders [53, 54], depression [55–57], and BPD [58, 59].

In spite of these encouraging findings various important research questions remain regarding emotion regulation. While concepts such as rumination and catastrophizing have been subsumed under the label of emotion regulation [1, 60], it is unclear if they should be conceptualized as cognitive components of the unregulated affect or even as symptoms of disorders caused by emotion dysregulation. Further, individuals might use more than one strategy to regulate their emotions, which interact to impact outcomes. Currently, many studies focus on only one aspect of emotion regulation (such as rumination, acceptance of emotions, cognitive restructuring, etc.) and hence do not allow analysis of interactions among various strategies.

**Fig 1:**
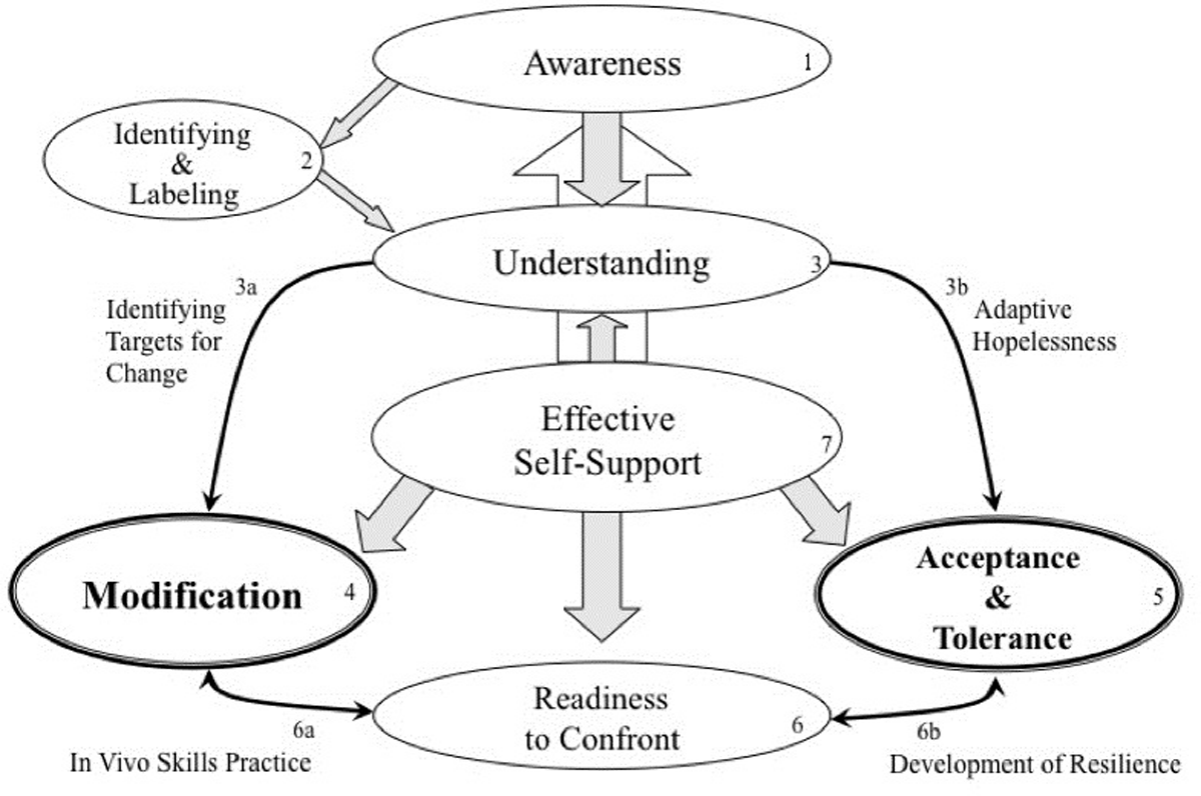
The Adaptive Coping with Emotions Model.

In order to answer these research questions and to utilize the notoriously broad concept of emotion regulation for clinical purposes, Berking and colleagues have developed the Adaptive Coping with Emotions (ACE) model [9, 61, 62] which is illustrated in Fig 1.

The ACE model synthesizes various other models of emotion or affect regulation [63–70] and conceptualizes adaptive emotion regulation as the situation-dependent interaction of nine skills that are commonly considered important elements of effective emotion regulation. The specific emotion-regulation skills include (1) the ability to be consciously aware of emotions, (2) the ability to identify and correctly label emotions, (3) the ability to identify what has caused and maintains one’s present emotions, (4) the ability to actively modify emotions in an adaptive manner or (5) the ability to accept and tolerate undesired emotions when they cannot be changed, (6) the ability to approach and confront situations likely to trigger negative emotions if this is necessary to attain personally relevant goals, and (7) the ability to provide effective self-support when working to cope with challenging emotions. According to the ACE model, unsuccessful emotion regulation occurs when individuals (a) try to apply emotion regulation skills but are unable to do so successfully, (b) have never developed these skills and thus are unable to even try to apply them, (c) have access to these skills but do not try to apply them. The model also includes the hypothesis that modification and acceptance/tolerance skills are the only skills in the model that are ultimately relevant for mental health. All other skills are themselves considered relevant only to the extent that they facilitate the successful use of modification or acceptance/tolerance.

To enable us to test the ACE model, we reviewed existing questionnaires on emotion regulation but were unable to identify instruments meeting all desired criteria. For example, some instruments include only a restricted scope of regulation strategies and did not cover a broad range of likely relevant skills [71–74]. Other questionnaires cover many of the skills included in the ACE model but still miss some skills that we consider important (e.g., the ability to understand what had cued one's present feelings is missing from the Difficulties in Emotion Regulation Scale [75]; acceptance-related skills are missing from the Trait Meta-Mood Scale [76]). Moreover, some instruments appear to use more items than necessary or desirable to assess comparably homogenous concepts from a methodological perspective and from the perceptive of clinical usability (e.g., [75]). Other questionnaires assess emotion regulation as part of a broader theoretical construct (such as experiential avoidance [77]) or assess concepts which should only be addressed as emotion regulation with great care in order to prevent the concept of emotion regulation from becoming too broad and losing its heuristic value (e.g., rumination: [78]; worrying: [79]; coping: [60]). To overcome these limitations and to obtain a clinically relevant and economic measure assessing a broad range of emotion regulation skills as described in the ACE model, we decided to develop a new self-report measure.

For this purpose, a pool of items was constructed first (in German) based on existing measures and on theoretical considerations for each of the skills of the ACE Model. Item characteristics of all items were then assessed in a study with 120 undergraduate students. For each skill, the three items with the highest item-subscale correlations were identified. The resulting measure (SEK-27: Selbsteinschätzung emotionaler Kompetenzen—27 [Self-report measure of emotional competencies—27 item version]) was then further evaluated in four subsequent studies with a community sample (N = 576) and three clinical samples (N_total_ = 238 patients). The measure was found to display good to excellent psychometric properties (e.g., cronbach’s α for the total scale = .90) as well as convincing evidence for its validity and sensitivity for change [80].

Subsequently, the SEK-27 was used in various studies on emotion regulation in the context of mental health. For example, it was found that emotion regulation skills as conceptualized in the ACE model significantly predicted subsequent symptoms of mental disorders over a 2-week and a 5-year interval [30, 34, 81]. Moreover, emotion regulation skills as assessed with the SEK-27 at the beginning of inpatient treatment for alcohol dependency predicted relapse during treatment, and emotion regulation assessed at the end of treatment predicted relapse during a 3-month follow-up [39]. Two other studies found out that some emotion regulation skills, assessed by the SEK-27, directly affect mental health whereas others only do so to the extent that they facilitate the successful use of the ultimately relevant skills accepting/tolerating [82]. Furthermore, the SEK-27 was used as outcome measure assessing changes in emotion regulation skills use in several studies to evaluate the efficacy of intense emotion regulation training. In the first study, it was found that police officers could significantly enhance their successful use of emotion regulation skills by participating the affect regulation training [9] from pre-treatment to post-treatment [83]. Additionally, it was shown in a sample of 289 participants meeting criteria for various disorders and in a sample of 432 patients meeting criteria for major depressive disorder that replacing parts of a cognitive-behavioral inpatient treatment with the affect regulation training enhances the treatment's efficacy, including a greater increase of successful emotion regulation skills use [55, 84]. Finally, it was shown that successful emotion regulation skills use, as measured by the SEK-27, predicted the subsequent reduction of depressive symptoms (but not vice versa) during inpatient treatment in a sample of 152 individuals meeting criteria for major depressive disorder [32].

When publishing these research findings, we received multiple requests from researchers and practitioners for an English version of the SEK-27. In order to comply with these requests and to enhance the international comparability of research findings on health-relevant effects of emotion regulation skills, we decided to comply with these requests and develop an English version of the SEK-27. We named this version the Emotion Regulation Skills Questionnaire (ERSQ). The primary aim of the present study was the development and validation of this measure. We expected, that the resulting measure would produce similar psychometric characteristics than the pre-existing German version.

## Method

### Development of the questionnaire

We developed the ERSQ by translating the German-based SEK-27 into English. Following the commonly used back-translation method [85–87], a bilingual expert in emotion regulation and clinical psychology first translated the SEK-27 from German into English. Then another bilingual expert who was blind with regard to the original measure re-translated the English version back to the original language. In a final step, both German Versions of the SEK-27 were compared to reveal and eliminate any arising inconsistencies. As no significant discrepancies between each item of both versions were identified, the English translation was accepted as the final English version.

This final version of the ERSQ consists of 27 items and is based on the emotion regulation skills defined by the ACE model [9]. Thus, successful skills use is assessed through the following nine subscales with three items per skill: *awareness* (e.g., “I paid attention to my feelings.”), *sensations* (e.g., “My physical sensations were a good indication of how I was feeling.”), *clarity* (e.g., “I was clear about what emotions I was experiencing.”), *understanding* (e.g., “I was aware of why I felt the way I felt.”), *modification* (e.g., “I was able to influence my negative feelings.”), *acceptance* (e.g., “I accepted my emotions.”), *tolerance* (e.g., “I felt I could tolerate my negative feelings.”), *readiness to confront distressing situations when necessary to attain personally relevant goals* (e.g., “I did what I had planned, even if it made me feel uncomfortable or anxious.”) and *self-support* (e.g., “I supported myself in emotionally distressing situations.”). Each item is assessed on a 5-point Likert-type scale (*0 = not at all* to *4 = almost always*), and preceded by the stem *“Last week I …”*. In addition to the subscales, a total score for successful emotion regulation can be computed as the average of all items.

### Participants and procedures

To evaluate the psychometric properties of the ERSQ, we first recruited a sample of 274 undergraduate students. After providing informed consent, participants completed a web-based survey at two different time points consisting of demographic questions, a set of published self-report questionnaires and the ERSQ. Overall, 274 students started to complete assessments and 263 participants successfully completed the entire survey. We used this later sample for all subsequent analyses. Participants’ average age was 20.6 years old (SD = 2.3, range: 18–39). About half of the sample (55%) was female. The majority (85%) was Caucasian, the remaining participants were African American (6%), and other Asian or Asian American (1%), East Indian (1%), Korean and Middle Eastern/Arab (0.5%), and other racial background (4%). About half of the sample (n = 119) completed the survey twice to provide data on retest-reliability. This subsample did not differ from the entire sample with regard to any of the socio demographic indicators. All study procedures followed internationally accepted human research guidelines such as the Helsinki Protocol. The ethics committee of Xavier University approved study procedures relevant for the validation in the student sample.

We additionally recruited a total of 35 patients from an inpatient psychiatric unit at an Australian hospital to test the hypothesis that ERSQ scores are higher in non-clinical than in clinical samples and to evaluate the sensitivity of the measure. Patients provided informed consent to have their de-identified questionnaire data used for research purposes, and then 27 patients completed a paper-pencil form of the ERSQ as part of the clinical standard assessment procedure at admission to inpatient treatment and 29 patients at discharge. Overall, 21 patients completed the ERSQ pre- and post treatment, 6 patients completed only pre-treatment and 8 only post-treatment. Age ranged from 19 to 52 years (*M* = 29.8; *SD* = 9.5) and 60% were female. Most frequent principal diagnosis was social phobia (22%), obsessive-compulsive disorder (22%), panic disorder (22%) and generalized anxiety disorder (19%). The majority of participants (70%) had a minimum of at least one additional comorbid diagnosis. All study procedures followed internationally accepted human research guidelines such as the Helsinki Protocol and were approved by the ethics committee of the clinic in which the study was conducted.

### Measures

To evaluate the convergent validity we tested the assumption that the ERSQ would be positively associated with other measures of emotion regulation but negatively with indicators of dysfunctional emotion regulation, loss of well-being and psychopathology. To assess these constructs participants completed the following questionnaires:

Emotion Regulation Questionnaire (ERQ; [72]). This questionnaire consists of 10 items and assesses individual differences in the emotion regulation strategies *expressive suppression* and *cognitive reappraisal*. The questionnaire demonstrated good internal consistency (in average, α = .79 for cognitive reappraisal and α = .73 for expressive suppression), and a 2-month test-retest reliability of about *r* = .7. The reported factor analyses supported the orthogonal two-factor structure of the measure [72].

Difficulties in Emotion Regulation Scale (DERS; [75]). The DERS contains 36 items to measure emotion-regulation difficulties on the six dimensions *non-acceptance of emotional responses*, *difficulties engaging in goal directed behavior*, *impulse control difficulties*, *lack of emotional awareness*, *limited access to effective emotion regulation strategies*, and *lack of emotional clarity*. The questionnaire has displayed adequate to high internal consistency (for the total score: α = .93; for all subscales α’s >.8), adequate 4–8 weeks test-retest reliability of *r* = .88 for the total scale and *r* = .69 (nonacceptance subscale) to *r* = .80 (clarity subscale) for the subscales. The authors further reported evidence for convergent and predictive validity and the reported factor analysis confirmed the six-factor structure [75].

Generalized Expectancy for Negative Mood Regulation Scale (NMR; [71]). This questionnaire consists of 30 items to assess expectancies for the self-regulation of negative moods. The NMR has high internal consistency (range for the total scale from α = .86 to α = .92), as well as adequate test–retest reliability over periods of 3–4 weeks (*r* = .74 for women; *r =* .76 for men) and 6–8 weeks (*r* = .78 for women; *r* = .67 for men). Furthermore, adequate construct and discriminant validity are reported for the NMR [71].

Toronto Alexithymia Scale (TAS-20; [88]) The TAS-20 is a 20-item scale to measure alexithymia on the three dimensions *difficulty identifying feelings*, *difficulty-describing feelings*, and *externally oriented thinking*. Parker, Taylor and Bagby [89] reported adequate internal consistency for the three TAS-20 factors (α > .70). The factorial validity was demonstrated by a confirmatory factor analysis that supported the three-factor model.

The Depression Anxiety Stress Scales (DASS; [90]). The DASS consists of 42 items assessing negative emotional symptoms on three dimensions: *Depression*, *Anxiety*, and *Stress*. Psychometric analyses of the DASS have provided strong evidence for the internal consistency (α = .91 for Depression; α = .81 for Anxiety; α = .89 for Stress) and convergent and discriminant validity of the three scales. Exploratory and confirmatory factor analyses of the DASS items supported the three-factor structure [91].

The Brief Symptom Inventory (BSI; [92]). The BSI contains 53 items to assess psychopathological symptoms on nine dimensions: *Somatization*, *Obsessive-compulsive symptoms*, *Interpersonal sensitivity*, *Depression*, *Anxiety*, *Hostility*, *Phobic anxiety*, *Paranoid ideation*, and *Psychoticism*. Derogatis and Melisaratos [92] reported good psychometric properties for the BSI with internal consistencies ranging from α = .71 (Psychoticism) to α = .85 (Depression) and test-retest-correlations ranging from *r =* .68 (Somatization) to *r =* .91 (Phobic Anxiety). The authors further report good evidence for convergent and construct validity.

### Data analysis

To evaluate the reliability of the ERSQ and its subscales we computed the internal consistency in terms of Cronbach’s Alpha within the student sample. Furthermore, test-retest reliability was evaluated by Pearson correlations of the ERSQ scores of two assessment points over a three-week interval within the student sample. To determine the dimensional validity we evaluated the factor structure of the ERSQ in the student sample, using exploratory factor analysis (EFA) to provide preliminary data on the factor structure and identify possibly underlying dimensions of emotion regulation assessed by our questionnaire. Moreover, confirmatory factor analysis (CFA) was used to test the proposed nine-factor structure of the ERSQ and to compare it to the results of the EFA and a general one-factor model. The Comparative Fit Index (CFI) and the Root Mean Square Error of Approximation (RMSEA) are used as indicators of model fit. By convention, excellence model fit is suggested by CFI values close to .95 and RMSEA values close to .06 [93], adequate fit by CFI values greater than .90 and RMSEA values less than .08, and acceptable fit by CFI values above .85 and RMSEA values less than .10 [93–95]. Furthermore, χ^2^ statistics are reported, despite their well-known sensitivity to large samples (e.g., [96]). Model comparisons were examined by using χ^2^ difference tests. To investigate the convergent validity, Pearson correlations between the ERSQ and other measures of emotion regulation and psychopathology were computed. To examine if the measurement was able to differentiate between mentally ill and healthy persons, as emotion regulation deficits are a widespread phenomenon within mental illnesses, we compared the mean ERSQ scores of students with those of individuals being treated for mental disorders. Therefore, independent t-tests were performed to detect significant differences between the ERSQ scores of both samples. Finally, to determine change sensitivity of the ERSQ, pre- and post treatment comparisons of the ERSQ scores of the clinical sample were examined by dependent t-tests. All calculations to evaluate reliability and validity, as well as the exploratory factor analysis were performed in SPSS 21. Confirmatory factor analysis was calculated in AMOS 21.

## Results

### Descriptive statistics and reliability

As shown in Table 1, mean scores of the ERSQ subscales within the student sample ranged between *M* = 2.45 (*SD* = 0.84; Scale Sensations) and *M* = 3.53 (*SD* = 0.84; Scale Self-support). The total scale score serving as overall indicator of successful emotion regulation skills use showed a mean score of *M* = 2.53 (*SD* = 0.68). As expected, the scales of the ERSQ showed moderate to strong intercorrelations (*r* = .47 − .87). Cronbach’s Alpha was calculated to determine the internal consistency of the ERSQ. As illustrated in Table 1, the ERSQ total score has a high internal consistency (α = .96). Moreover, all of the ERSQ subscales displayed acceptable to good internal consistency, with Cronbach's Alphas ranging from α = .73 (self-support) to α = .88 (tolerance). As expected for a measure developed to assess a semi stable construct and to be sensitive to change, the test-retest reliability for all scales over a mean period of 3 weeks was comparatively low (r_tt_ = .30 − .60). Item statistics are displayed in Table 2, with corrected item-total correlations ranging from r_it_ = .49 − .77 and medium to easy item difficulties (*p* = 0.56–0.68), both indicating acceptable to good psychometric item properties.

**Table 1:** Descriptive statistics, internal consistency, retest-reliability and intercorrelations (Pearson) of the ERSQ scales.

Scale	M	SD	α	r_tt_	1	2	3	4	5	6	7	8	9	10
	(N = 263)			(N = 112)										
Awareness	2.52	0.81	.76	.60***	-	-	-	-	-	-	-	-	-	-
Sensations	2.45	0.84	.75	.49***	.84***	-	-	-	-	-	-	-	-	-
Clarity	2.57	0.86	.85	.43***	.87***	.82***	-	-	-	-	-	-	-	-
Understanding	2.62	0.83	.85	.48***	.84***	.76***	.85***	-	-	-	-	-	-	-
Acceptance	2.55	0.83	.79	.53***	.84***	.61***	.67***	.62***	-	-	-	-	-	-
Tolerance	2.46	0.91	.88	.59***	.79***	.56***	.55***	.49***	.77***	-	-	-	-	-
Confrontation	2.66	0.80	.77	.46**	.72***	.47***	.47***	.49***	.62***	.59***	-	-	-	-
Self-support	2.53	0.79	.73	.30***	.79***	.58***	.60***	.56***	.63***	.60***	.61***	-	-	-
Modification	2.37	0.79	.74	.47***	.84***	.61***	.65***	.62***	.72***	.70***	.59***	.66***	-	-
ERSQ total	2.53	0.68	.96	.59***	.82***	.84***	.87***	.84***	.84***	.79***	.72***	.79***	.84***	-

α = Cronbach’s Alpha; r_tt_ = Retest-Reliability;

**p< .001

***p< .0001. All intercorrelations are significant at *p*< .0001

**Table 2:** Mean, standard deviation, item difficulty and corrected item total-correlation for the items composing the ERSQ (N = 265).

Item	M	SD	Item Difficulty p-value	Corrected item-total correlation
1	2.59	1.02	0.64	0.63
2	2.44	0.95	0.61	0.62
3	2.54	0.94	0.63	0.74
4	2.38	1.00	0.59	0.70
5	2.39	0.98	.059	0.70
6	2.50	0.95	0.62	0.71
7	2.45	1.03	0.61	0.73
8	2.59	1.06	0.64	0.49
9	2.63	0.91	0.65	0.60
10	2.24	0.98	0.56	0.62
11	2.58	0.97	0.64	0.75
12	2.33	1.02	0.58	0.61
13	2.57	0.96	0.64	0.71
14	2.33	1.08	0.58	0.58
15	2.43	1.00	0.60	0.57
16	2.65	0.97	0.66	0.53
17	2.52	1.00	0.63	0.63
18	2.45	1.05	0.61	0.67
19	2.63	0.92	0.65	0.69
20	2.75	0.92	0.68	0.66
21	2.43	0.99	0.60	0.70
22	2.73	0.96	0.68	0.61
23	2.73	0.97	0.68	0.72
24	2.57	0.98	0.64	0.66
25	2.64	1.02	0.65	0.77
26	2.55	1.00	0.63	0.66
27	2.54	0.96	0.63	0.70

N = 263.

### Factor structure

A principal component factor analysis was conducted on the 27 items with oblique factor rotation (direct oblimin) to allow for correlations among factors. The Kaiser-Meyer-Olkin measure verified the sampling adequacy (KMO = .95) and Bartlett’s test of sphericity indicated sufficiently large correlations between items (χ^2^ (351) = 4902.81, p < .0001). The analysis revealed three factors with eigenvalues > 1 (Kaiser-Guttman criterion) which in combination explained 61.07% of the variance. As shown in Table 3, item communalities ranged from .45 to .78. Overall, the hypothesized scales understanding, clarity, awareness and body sensations loaded on the first factor (eigenvalue: 12.9), the scales tolerance, acceptance and regulation on the second (eigenvalue: 2.3), and the third factor (eigenvalue: 1.3) contains the subscales confrontation and self-support. There were four items with double loadings on two factors above .30

**Table 3:** Principal component analysis with oblique factor rotation.

Items	Scale	Comunality (h^2^)	Factorloadings		
			1	2	3
11	Understanding	0.78	**.91**	-.01	-.04
1	Awareness	0.65	**.88**	-.14	-.01
13	Clarity	0.69	**.82**	.07	-.06
6	Clarity	0.67	**.77**	.15	-.10
3	Understanding	0.68	**.75**	.15	-.05
20	Understanding	0.63	**.75**	-.13	.20
14	Sensations	0.50	**.72**	-.08	.06
25	Clarity	0.69	**.71**	.13	.08
7	Sensations	0.68	**.71**	.29	-.18
19	Awareness	0.60	**.67**	.00	.18
24	Sensations	0.51	**.58**	.12	.11
12	Awareness	0.45	**.56**	.06	.14
26	Tolerance	0.75	-.06	**.90**	.00
18	Tolerance	0.72	-.01	**.87**	-.02
4	Tolerance	0.70	.07	**.79**	.01
17	Acceptance	0.61	.01	**.76**	.03
5	Acceptance	0.67	.23	**.72**	-.18
27	Self support	0.63	.08	**.55**	**.31**
2	Modification	0.47	.25	**.52**	.00
10	Modification	0.49	.09	**.48**	.26
23	Acceptance	0.59	.26	**.41**	.27
21	Modification	0.54	**.34**	**.37**	.19
8	confront	0.58	.04	.01	**.74**
9	Self support	0.67	.20	-.04	**.73**
15	Self support	0.48	.25	.04	**.52**
16	confront	0.48	-.07	**.36**	**.50**
22	confront	0.61	-.09	**.49**	**.49**

N = 263; factor loadings < .30 are in bold face.

Furthermore, a confirmatory factor analysis was conducted to test the fit of the data to the theoretically proposed nine dimensions of emotion regulation, and to compare this theoretically-based model of the ERSQ with (a) the three factor solution found in the exploratory factor analysis and (b) the most parsimonious single-factor solution. As indicated in Table 4, the nine-factor model showed an adequate fit to the data (CFI = .90; RMSEA = .07), whereas the three-factor model ranged from poor to adequate fit (CFI = .88; RMSEA = .08) and the single-factor model showed a poor model fit (CFI = .75; RMSEA = .12). Moreover, the chi-square difference tests demonstrated that the nine-factor model showed a significantly better fit than the three-factor model (Δχ2 = 77.94, *df* = 10, *p*< .001) and the single-factor model (Δχ2 = 708.04, *df* = 35, *p*< .001).

**Table 4:** Measures of global fit for all models estimated.

Models	χ2	df	p	CFI	RMSEA
one-factor model	1489.11	324	< .001	.75	.12
three-factor model	859.01	299	< .001	.88	.08
nine-factor model	781.07	289	< .001	.90	.07

### Validity

Table 5 shows the correlations between the ERSQ and other measures of emotion regulation and psychopathology. With regard to the association with other ER measures, it was found that the ERSQ subscales and the ERSQ total score were constantly positive correlated with the ERQ reappraisal scale and negative correlated with the ERQ suppression scale. Furthermore, the NMR total score was significantly correlated with all ERSQ scales ranging from *r* = .35 (Awareness) to *r* = .56 (Acceptance and Modification). Regarding the correlations with the DERS, we found an even more differentiated pattern of correlations. The ERSQ awareness subscale is strongly negative correlated with the DERS awareness subscale (*r* = -.67; *p*< .0001), as well as both clarity subscales (*r* = -.57; *p*< .0001) and the ERSQ Acceptance and DERS Non-Acceptance subscales (*r* = -.45; *p* < .0001). As expected, the DERS Strategies subscale showed stronger correlations with the acceptance, tolerance, readiness to confront, self-support and modification subscales (*r* = -.41 − -.50; *p*< .0001) than with the awareness, sensations, clarity and understanding subscales (*r* = -.25 − -.36; *p*< .0001).

**Table 5:** Correlations with other measures (Pearson).

Scales	ERQ-R	ERQ-S	DERS-A	DERS-C	DERS-NA	DERS-S	NMR	TAS	DASS-S	DASS-D	DASS-A	BSI ^1^
Awareness	.25***	-.33***	-.67***	-.48***	-.17**	-.25***	.35***	-.45***	-.19**	-.14*	-.17**	-.18**
Sensations	.26***	-.27***	-.63***	-.52***	-.25***	-.32***	.41***	-.48***	-.31***	-.26***	-.23***	-.30***
Clarity	.25***	-.33***	-.62***	-.57***	-.33***	-.36***	.47***	-.53***	-.34***	-.25***	-.29***	-.31***
Understanding	.28***	-.36***	-.69***	-.61***	-.34***	-.35***	.40***	-.53***	-.29***	-.23***	-.25***	-.25***
Acceptance	.31***	-.28***	-.45***	-.54***	-.45***	-.50***	.56***	-.49***	-.48***	-.42***	-.34***	-.44***
Tolerance	.30***	-.11*	-.30***	-.40***	-.33***	-.49***	.50***	-.37***	-.45***	-.41***	-.26***	-.41***
Readiness to confront	.26***	-.19**	-.33***	-.38***	-.21***	-.45***	.42***	-.38***	-.34***	-.32***	-.25***	-.32***
Self-support	.42***	-.24***	-.44***	-.40***	-.26***	-.41***	.50***	-.45***	-.30***	-.28***	-.21***	-.29***
Modification	.36***	-.24***	-.46***	-.52***	-.33***	-.50***	.56***	-.48***	-.41***	-.40***	-.27***	-.38***
ERSQ total score	.36***	-.32***	-.62***	-.60***	-.36***	-.50***	.57***	-.57***	-.43***	-.37***	-.31***	-.39***

ERQ-R = Emotion Regulation Scale-Reappraisal; ERQ-S = Emotion Regulation Scale-Suppression; DERS-A = Difficulties in Emotion Regulation Scale-Awareness; DERS-C = Difficulties in Emotion Regulation Scale-Clarity; DERS-NA = Difficulties in Emotion Regulation Scale-NonAcceptance; DERS-S = Difficulties in Emotion Regulation Scale-Strategies; NMR = Negative Mood Regulation Scale; TAS = Toronto Alexithymia Scale; DASS-S = Depression Anxiety Stress Scales–Stress; DASS-D = Depression Anxiety Stress Scales–Depression; DASS-S = Depression Anxiety Stress Scales–Anxiety; BSI = Brief Symptom Inventory (Global Severity Index; GSI).

*p< .05

**p> .01

***p< .0001.

^1^ The first author can obtain results for subscales of all measures.

With regard to the associations between the ERSQ and indicators of mental health and mental disorders, it was found that all ERSQ subscales and the ERSQ total score are negatively correlated with measures of psychopathology. Overall, the awareness subscale showed the weakest correlations with DASS subscales and BSI (*r* = -.14 − -0.19; *p*s > 0.05) and acceptance showed the strongest correlations (*r* = -.34 − -.48; *p* < .0001). The TAS-20 scale score correlated especially strong with the ERSQ subscales clarity and understanding (for both: *r* = -.53; *p* < .0001).

To evaluate if the ERSQ could differentiate between different groups, we compared the ERSQ scores of students (time 1) with those of the clinical sample (at admission). Results are presented in Table 6. The two groups differed significantly according to the subscales acceptance (*t*(289) = 3.87; *p* < .001; *d* = 0.76), modification (*t*(289) = 3.40; *p* < .001; *d* = 0.65), readiness to confront (*t*(289) = 2.55; *p* < .01; *d* = 0.46), and tolerance (*t*(289) = 1.78; *p* < .05; *d* = 0.37) indicating that these scales differentiate between the two samples. The total scale score failed to reach the level of statistical significance by a small margin (*t*(289) = 1.57; *p =* 0.059; *d* = 0.32).

**Table 6:** Means and standard deviations for the clinical sample for admission and discharge, comparison of clinical and non-clinical sample and comparisons of clinical sample pre and post treatment of the ERSQ Scales.

	CS-admission		CS-discharge		Comparison CS and NCS				CS—Comparison pre post treatment			
	(*N* = 27)		(*N* = 29)						(N = 21)			
Scale	*M*	*SD*	*M*	*SD*	*M*_*DIFF*_	*t*	*df*	*d*	*M*_*t2-t1*_	*t*	*df*	*d*
Awareness	2.60	0.87	2.99	0.84	-0.08	-.49	289	-0.10	0.44	-2.45*	20	0.69
Sensations	2.54	0.92	3.99	0.78	-0.09	-.51	289	-0.10	0.38	-2.41*	20	0.28
Clarity	2.65	0.84	3.18	0.70	-0.09	-.51	289	-0.09	0.43	-3.34**	20	0.43
Understanding	2.63	0.83	3.09	0.73	-0.01	-.05	289	-0.01	0.52	-3.10**	20	0.51
Acceptance		0.88	2.84	0.70	0.64	3.87**	289	0.76	1.11	-5.03**	20	0.48
Tolerance		0.80	2.85	0.66	0.32	1.78*	289	0.37	0.86	-4.57**	20	0.61
Readiness to confront	2.24	1.02	3.06	0.85	0.41	2.55**	289	0.46	0.71	-3.42**	20	0.62
Self-support	2.29	0.95	2.76	0.98	0.25	1.53	289	0.27	0.59	-2.65**	20	0.31
Modification	1.83	0.88	2.60	0.97	0.54	3.40**	289	0.65	0.98	-4.27**	20	0.33
ERSQ total	2.32	0.64	2.93	0.64	0.21	1.57	289	0.32	0.67	-4.66**	20	0.65

Note: CS = Clinical Sample; NCS = Non-Clinical Sample:

*p< .05,

**p< .01

To evaluate the sensitivity for change of the ERSQ, we compared the ERSQ scores of the clinical sample at admission and discharge. As indicated in Table 5, ERSQ scores significantly increased for the total score (*t*(20) = -4.66; *p* < .01) and all subscales during treatment (awareness: *t*(20) = -2.45; *p* < .05; sensations: *t*(20) = -2.41; *p* < .05; clarity: *t*(20) = -3.34; *p* < .01; understanding: *t*(20) = -3.10; *p* < .01; acceptance: *t*(20) = -5.03; *p* < .01; tolerance: *t*(20) = -4.57; *p* < .01; readiness to confront: *t*(20) = -3.42; *p* < .01; self-support: *t*(20) = -2.65; *p* < .01; modification: *t*(20) = -4.27; *p* < .01). Effect sizes for these comparisons range between *d* = 0.28–0.69.

## Discussion

The aim of the current study was to develop and validate an English version of the SEK-27 for both clinical and research purposes. Therefore, the ERSQ (originally in German language) was translated into English language and validated in a student and a clinical sample. Findings indicate that the ERSQ displays adequate to good psychometric properties and can be used as a short, reliable and valid instrument simultaneously assessing a broad range of emotion regulation skills.

Findings from the present study are consistent with previous research [39, 80–83] as both the total score as well as all subscales of the ERSQ show good to excellent internal reliability and a moderate stability which implies that the scale can be used to asses change occurring over time. Also consistent with research on the German version [80], the confirmatory factor analyses supported the postulated nine-factor structure of the measure, suggesting that the separated assessment of the nine postulated dimensions is reasonable.

It is of note that the exploratory factor analysis suggested a three-factor solution. However, the significant item and sub-scale intercorrelations responsible for this finding likely result from different emotion regulation skills reciprocally affecting each other (e.g., [82]). Therefore, a fewer factor solution is no clear evidence against the distinction between sub-skills. Evidence for this hypothesis comes from the confirmatory factor analyses of the present study as well as from the differences across ERSQ subscales with regard to the associations with other measures and with regard to the comparisons between the non-clinical and the clinical sample.

The pattern of associations between the ERSQ scales and instruments focusing on emotion regulation provides significant evidence for the validity of the ERSQ. For example, similar subscales of different instruments (e.g., ERSQ Acceptance and DERS Non-Acceptance, ERSQ Awareness and DERS Awareness subscales) show considerable stronger correlations than distinct subscales. The TAS-20 [88] which measures a concept of alexithymia on the three dimensions *difficulty identifying feelings*, *difficulty describing feelings*, and *externally-oriented thinking*, correlated most strongly with the ERSQ subscales clarity and understanding, on which especially alexithymia-prone individuals would be expected having difficulties. Further evidence for the construct validity of the ERSQ comes from the strong negative associations of the ERSQ with validated measures of psychopathology [88, 90–92]. The finding that the ERSQ subscales acceptance, tolerance and modification are most strongly associated with the indicators of mental health is consistent with the hypothesis that these skills are the skills ultimately important for mental health [9, 61, 82]. Additionally, evidence for the validity of the ERSQ comes from the findings that several subscales—acceptance, tolerance, readiness to confront and modification—of the measure differentiate between the non-clinical and the clinical sample. Somewhat unexpectedly, the ERSQ could not differentiate between the two samples through the total score and the other subscales. Potentially, this finding can be explained by assuming that mental health problems are highly prevalent among university students [97]. Finally, consistent with findings on the German version [80] the ERSQ displayed significant evidence for sensitivity of change in the context of treatment for mental disorders.

There are several limitations to our study. Major limitations include (a) the exclusive use of self-report measures to assess discriminant and concordant constructs, (b) the use of a convenience sample of students who have not been screened for the absence of mental disorders, and (c) the small sample size of the clinical sample which was recruited in another country than the student sample. Therefore, future studies should further scrutinize the validity of the ERSQ with the help of observer-based [98, 99], experimental [100], or biological [101, 102] indicators of ER (and mental health). Furthermore, such studies should systematically compare findings from samples unambiguously identified as healthy with those from (large) clinical samples (ideally in various countries to further clarify the importance of cultural differences according to ER; for further information on this issue see [103]). Additionally, it is of note that the ERSQ (and many other measure of emotion regulation) assesses how participants respond to their “feelings” in general without discriminating between different emotions (or in general affective states including stress responses, moods or even motivational impulses, see [3]). Therefore, it is unclear what kind of feelings participants referred to when completing the ERSQ (or similar measures). For example, a depressed patient might refer to his or her feelings of sadness and despair when completing the ERSQ whereas patients exclusively suffering from an anxiety disorder may refer to his or her fears and states of anxiety. To overcome this limitation, we have recently developed an affect specific version of the ERSQ. The modified version of the ERSQ separately assesses the ability to respond to five affective states that appear particularly relevant for clinical research and practice (stress/tension, anxiety, sadness, anger and depressed mood) as well as positive emotions. The German version of this questionnaire indicates good reliability and validity. It further provides strong evidence that the abilities to cope with different affective states have both a general ER skills component and an affect-specific component [104]. To our knowledge, no emotion specific questionnaire exists in English, thus future research should develop and validate an English version of this emotion-specific version of the ERSQ.

## Supporting information

S1 File Minimal data set clinical sample. (SAV)

S2 File Minimal data set student sample. (SAV)

## References

[1] Aldao A, Nolen-Hoeksema S, Schweizer S. Emotion-regulation strategies across psychopathology: A meta-analytic review. Clin Psychol Rev. 2010;30(2):217–37. 10.1016/j.cpr.2009.11.004 20015584

[2] Berking M, Wupperman P. Emotion regulation and mental health: recent findings, current challenges, and future directions. Curr Opin Psychiatry. 2012;25(2):128–34. 10.1097/YCO.0b013e3283503669 22262030

[3] Gross JJ. Handbook of emotion regulation. Second Edition. ed. New York: The Guilford Press; 2014 xviii, 669 pages p.

[4] Berenbaum H, Raghavan C, Le H-N, Vernon LL, Gomez JJ. A Taxonomy of Emotional Disturbances. Clinical Psychology: Science and Practice. 2003;10(2):206–26.

[5] Greenberg LS. Integrating an emotion-focused approach to treatment into psychotherapy integration. Journal of Psychotherapy integration. 2002;12(2):154.

[6] Kring AM, Bachorowski J-A. Emotions and Psychopathology. Cognition and Emotion. 1999;13(5):575–99.

[7] Mennin D, Farach F. Emotion and Evolving Treatments for Adult Psychopathology. Clinical Psychology: Science and Practice. 2007;14(4):329–52.

[8] Thompson RA. Emotion regulation: a theme in search of definition. Monogr Soc Res Child Dev. 1994;59(2–3):25–52. 7984164

[9] Berking M, Whitley B. Affect Regulation Training. New York: Springer; 2014.

[10] Hayes SC, Wilson KG, Gifford EV, Follette VM, Strosahl K. Experimental avoidance and behavioral disorders: a functional dimensional approach to diagnosis and treatment. J Consult Clin Psychol. 1996;64(6):1152–68. 8991302 10.1037//0022-006x.64.6.1152

[11] Catanzaro SJ, Wasch HH, Kirsch I, Mearns J. Coping‐related expectancies and dispositions as prospective predictors of coping responses and symptoms. Journal of Personality. 2000;68(4):757–88. 10934689 10.1111/1467-6494.00115

[12] Ehring T, Fischer S, Schnülle J, Bösterling A, Tuschen-Caffier B. Characteristics of emotion regulation in recovered depressed versus never depressed individuals. Personality and Individual Differences. 2008;44(7):1574–84.

[13] Kassel JD, Bornovalova M, Mehta N. Generalized expectancies for negative mood regulation predict change in anxiety and depression among college students. Behaviour Research & Therapy. 2007;45(5):939–50. 17010932 10.1016/j.brat.2006.07.014

[14] Baker R, Holloway J, Thomas PW, Thomas S, Owens M. Emotional processing and panic. Behav Res Ther. 2004;42(11):1271–87. 10.1016/j.brat.2003.09.002 15381438

[15] Turk CL, Heimberg RG, Luterek JA, Mennin DS, Fresco DM. Emotion dysregulation in generalized anxiety disorder: A comparison with social anxiety disorder. Cognitive Therapy Research. 2005;29(1):89–106.

[16] Tull MT, Barrett HM, McMillan ES, Roemer L. A preliminary investigation of the relationship between emotion regulation difficulties and posttraumatic stress symptoms. BEHAV THER. 2007;38(3):303–13. 10.1016/j.beth.2006.10.001 17697854

[17] Linehan M. Cognitive-behavioral treatment of borderline personality disorder. New York: Guilford Press; 1993 xvii, 558 p. p.

[18] Lynch TR, Cheavens JS, Cukrowicz KC, Thorp SR, Bronner L, Beyer J. Treatment of older adults with co-morbid personality disorder and depression: a dialectical behavior therapy approach. Int J Geriatr Psychiatry. 2007;22(2):131–43. 10.1002/gps.1703 17096462

[19] Selby EA, Fehling KB, Panza EA, Kranzler A. Rumination, mindfulness, and borderline personality disorder symptoms. Mindfulness. 2016;7(1):228–35.

[20] Bydlowski S, Corcos M, Jeammet P, Paterniti S, Berthoz S, Laurier C, et al Emotion‐processing deficits in eating disorders. International Journal of Eating Disorders. 2005;37(4):321–9. 10.1002/eat.20132 15856501

[21] Harrison A, Sullivan S, Tchanturia K, Treasure J. Emotion recognition and regulation in anorexia nervosa. Clin Psychol Psychother. 2009;16(4):348–56. 10.1002/cpp.628 19517577

[22] Whiteside U, Chen E, Neighbors C, Hunter D, Lo T, Larimer M. Difficulties regulating emotions: Do binge eaters have fewer strategies to modulate and tolerate negative affect? Eating Behaviors. 2007;8(2):162–9. 10.1016/j.eatbeh.2006.04.001 17336786

[23] Lavender JM, Wonderlich SA, Engel SG, Gordon KH, Kaye WH, Mitchell JE. Dimensions of emotion dysregulation in anorexia nervosa and bulimia nervosa: A conceptual review of the empirical literature. Clin Psychol Rev. 2015;40:111–22. 10.1016/j.cpr.2015.05.010 26112760 PMC4537813

[24] Fox HC, Axelrod SR, Paliwal P, Sleeper J, Sinha R. Difficulties in emotion regulation and impulse control during cocaine abstinence. Drug Alcohol Depend. 2007;89(2–3):298–301. 10.1016/j.drugalcdep.2006.12.026 17276626

[25] Fox HC, Hong KA, Sinha R. Difficulties in emotion regulation and impulse control in recently abstinent alcoholics compared with social drinkers. Addict Behav. 2008;33(2):388–94. 10.1016/j.addbeh.2007.10.002 18023295

[26] Ghorbani F, Khosravani V, Sharifi Bastan F, Jamaati Ardakani R. The alexithymia, emotion regulation, emotion regulation difficulties, positive and negative affects, and suicidal risk in alcohol-dependent outpatients. Psychiatry research. 2017;252:223–30. 10.1016/j.psychres.2017.03.005 28285249

[27] Shaw P, Stringaris A, Nigg J, Leibenluft E. Emotion dysregulation in attention deficit hyperactivity disorder. Am J Psychiatry. 2014;171(3):276–93. 10.1176/appi.ajp.2013.13070966 24480998 PMC4282137

[28] Van Rheenen TE, Murray G, Rossell SL. Emotion regulation in bipolar disorder: profile and utility in predicting trait mania and depression propensity. Psychiatry research. 2015;225(3):425–32. 10.1016/j.psychres.2014.12.001 25537486

[29] Aldao A, Nolen-Hoeksema S. When are adaptive strategies most predictive of psychopathology? J Abnorm Psychol. 2012;121(1):276–81. 10.1037/a0023598 21553934

[30] Berking M, Wirtz CM, Svaldi J, Hofmann SG. Emotion regulation predicts symptoms of depression over five years. Behav Res Ther. 2014;57:13–20. 10.1016/j.brat.2014.03.003 24754907

[31] Kraaij V, Pruymboom E, Garnefski N. Cognitive coping and depressive symptoms in the elderly: a longitudinal study. Aging Ment Health. 2002;6(3):275–81. 10.1080/13607860220142387 12217096

[32] Radkovsky A, McArdle JJ, Bockting CL, Berking M. Successful emotion regulation skills application predicts subsequent reduction of symptom severity during treatment of major depressive disorder. J Consult Clin Psychol. 2014;82(2):248–62. 10.1037/a0035828 24564219

[33] Hino T, Takeuchi T, Yamanouchi N. A 1-year follow-up study of coping in patients with panic disorder. Compr Psychiatry. 2002;43(4):279–84. 12107865 10.1053/comp.2002.33495

[34] Wirtz CM, Hofmann SG, Riper H, Berking M. Emotion regulation predicts anxiety over a five-year interval: a cross-lagged panel analysis. Depress Anxiety. 2014;31(1):87–95. 10.1002/da.22198 24151095

[35] Tragesser SL, Solhan M, Brown WC, Tomko RL, Bagge C, Trull TJ. Longitudinal associations in borderline personality disorder features: Diagnostic Interview for Borderlines-Revised (DIB-R) scores over time. J Pers Disord. 2010;24(3):377–91. 10.1521/pedi.2010.24.3.377 20545501 PMC4157937

[36] Tragesser SL, Solhan M, Schwartz-Mette R, Trull TJ. The role of affective instability and impulsivity in predicting future BPD features. J Pers Disord. 2007;21(6):603–14. 10.1521/pedi.2007.21.6.603 18072862

[37] Anestis MD, Selby EA, Crosby RD, Wonderlich SA, Engel SG, Joiner TE. A comparison of retrospective self-report versus ecological momentary assessment measures of affective lability in the examination of its relationship with bulimic symptomatology. Behav Res Ther. 2010;48(7):607–13. 10.1016/j.brat.2010.03.012 20392437 PMC2878857

[38] Hilbert A, Tuschen-Caffier B. Maintenance of binge eating through negative mood: A naturalistic comparison of binge eating disorder and bulimia nervosa. International Journal of Eating Disorders. 2007;40(6):521–30. 10.1002/eat.20401 17573697

[39] Berking M, Margraf M, Ebert D, Wupperman P, Hofmann SG, Junghanns K. Deficits in emotion-regulation skills predict alcohol use during and after cognitive-behavioral therapy for alcohol dependence. J Consult Clin Psychol. 2011;79(3):307–18. 10.1037/a0023421 21534653 PMC3109184

[40] Ehring T, Tuschen-Caffier B, Schnülle J, Fischer S, Gross JJ. Emotion regulation and vulnerability to depression: spontaneous versus instructed use of emotion suppression and reappraisal. Emotion. 2010;10(4):563–72. 10.1037/a0019010 20677873

[41] Tull MT, Roemer L. Emotion regulation difficulties associated with the experience of uncued panic attacks: evidence of experiential avoidance, emotional nonacceptance, and decreased emotional clarity. BEHAV THER. 2007;38(4):378–91. 10.1016/j.beth.2006.10.006 18021952

[42] Westermann S, Kesting ML, Lincoln TM. Being deluded after being excluded? How emotion regulation deficits in paranoia-prone individuals affect state paranoia during experimentally induced social stress. Behav Ther. 2012;43(2):329–40. 10.1016/j.beth.2011.07.005 22440069

[43] Lynch TR, Trost WT, Salsman N, Linehan MM. Dialectical behavior therapy for borderline personality disorder. Annu Rev Clin Psychol. 2007;3:181–205. 10.1146/annurev.clinpsy.2.022305.095229 17716053

[44] Harley R, Sprich S, Safren S, Jacobo M, Fava M. Adaptation of dialectical behavior therapy skills training group for treatment-resistant depression. J Nerv Ment Dis. 2008;196(2):136–43. 10.1097/NMD.0b013e318162aa3f 18277222

[45] Lynch TR, Morse JQ, Mendelson T, Robins CJ. Dialectical behavior therapy for depressed older adults: a randomized pilot study. Am J Geriatr Psychiatry. 2003;11(1):33–45. 12527538

[46] Linehan MM, Dimeff LA, Reynolds SK, Comtois KA, Welch SS, Heagerty P, et al Dialectical behavior therapy versus comprehensive validation therapy plus 12-step for the treatment of opioid dependent women meeting criteria for borderline personality disorder. Drug Alcohol Depend. 2002;67(1):13–26. 12062776 10.1016/s0376-8716(02)00011-x

[47] Massah O, Sohrabi F, A'Azami Y, Doostian Y, Farhoudian A, Daneshmand R. Effectiveness of Gross Model-Based Emotion Regulation Strategies Training on Anger Reduction in Drug-Dependent Individuals and its Sustainability in Follow-up. Int J High Risk Behav Addict. 2016;5(1):e24327 10.5812/ijhrba.24327 27162759 PMC4859936

[48] Safer DL, Telch CF, Agras WS. Dialectical behavior therapy for bulimia nervosa. Am J Psychiatry. 2001;158(4):632–4. 10.1176/appi.ajp.158.4.632 11282700

[49] Telch CF, Agras WS, Linehan MM. Dialectical behavior therapy for binge eating disorder. J Consult Clin Psychol. 2001;69(6):1061–5. 11777110 10.1037//0022-006x.69.6.1061

[50] Cloitre M, Koenen KC, Cohen LR, Han H. Skills training in affective and interpersonal regulation followed by exposure: a phase-based treatment for PTSD related to childhood abuse. J Consult Clin Psychol. 2002;70(5):1067–74. 12362957 10.1037//0022-006x.70.5.1067

[51] Varkovitzky RL, Sherrill AM, Reger GM. Effectiveness of the Unified Protocol for Transdiagnostic Treatment of Emotional Disorders Among Veterans With Posttraumatic Stress Disorder: A Pilot Study. Behav Modif. 2018;42(2):210–30. 10.1177/0145445517724539 28845680

[52] Mennin DS. Emotion regulation therapy for generalized anxiety disorder. Clinical Psychology & Psychotherapy. 2004;11(1):17–29.

[53] Clyne C, Latner JD, Gleaves DH, Blampied NM. Treatment of emotional dysregulation in full syndrome and subthreshold binge eating disorder. Eat Disord. 2010;18(5):408–24. 10.1080/10640266.2010.511930 20865594

[54] Corstorphine E. Cognitive–emotional–behavioural therapy for the eating disorders: Working with beliefs about emotions. European Eating Disorders Review: The Professional Journal of the Eating Disorders Association. 2006;14(6):448–61.

[55] Berking M, Ebert D, Cuijpers P, Hofmann SG. Emotion regulation skills training enhances the efficacy of inpatient cognitive behavioral therapy for major depressive disorder: a randomized controlled trial. Psychother Psychosom. 2013;82(4):234–45. 10.1159/000348448 23712210

[56] Ellison JA, Greenberg LS, Goldman RN, Angus L. Maintenance of gains following experiential therapies for depression. J Consult Clin Psychol. 2009;77(1):103–12. 10.1037/a0014653 19170457

[57] Elices M, Soler J, Feliu-Soler A, Carmona C, Tiana T, Pascual JC, et al Combining emotion regulation and mindfulness skills for preventing depression relapse: a randomized-controlled study. Borderline Personal Disord Emot Dysregul. 2017;4:13 10.1186/s40479-017-0064-6 28690851 PMC5497384

[58] Gratz KL, Gunderson JG. Preliminary data on an acceptance-based emotion regulation group intervention for deliberate self-harm among women with borderline personality disorder. Behav Ther. 2006;37(1):25–35. 10.1016/j.beth.2005.03.002 16942958

[59] Sauer-Zavala S, Bentley KH, Wilner JG. Transdiagnostic Treatment of Borderline Personality Disorder and Comorbid Disorders: A Clinical Replication Series. J Pers Disord. 2016;30(1):35–51. 10.1521/pedi_2015_29_179 25710737

[60] Garnefski N, Kraaij V. The cognitive emotion regulation questionnaire. European Journal of Psychological Assessment. 2007;23(3):141–9.

[61] Berking M. Training emotionaler Kompetenzen. Heidelberg: Springer-Verlag; 2008.

[62] Berking M, Schwarz J. Affect regulation training In: J. GJ, editor. Handbook of emotion regulation, 2nd ed New York, NY, US: Guilford Press; 2014 p. 529–47.

[63] Eisenberg N. Emotion, regulation, and moral development. Annu Rev Psychol. 2000;51:665–97. 10.1146/annurev.psych.51.1.665 10751984

[64] Gottman JM, Katz LF. Effects of marital discord on young children's peer interaction and health. Developmental psychology. 1989;25(3):373.

[65] Gross JJ. The emerging field of emotion regulation: an integrative review. Review of general psychology. 1998;2(3):271.

[66] Larsen RJ. Toward a science of mood regulation. Psychological Inquiry. 2000;11(3):129–41.

[67] Lazarus RS. Progress on a cognitive-motivational-relational theory of emotion. Am Psychol. 1991;46(8):819–34. 1928936 10.1037//0003-066x.46.8.819

[68] Leahy RL. A model of emotional schemas. Cognitive and Behavioral Practice. 2002;9(3):177–90.

[69] Saarni C. The development of emotional competence. New York u.a 1999.

[70] Salovey P, Mayer JD. Emotional intelligence. Imagination, cognition, and Personality. 1990;9(3):185–211.

[71] Catanzaro SJ, Mearns J. Measuring generalized expectancies for negative mood regulation: Initial scale development and implications. Journal of Personality Assessment. 1990;54(3–4):546–63. 10.1080/00223891.1990.9674019 2348341

[72] Gross JJ, John OP. Individual differences in two emotion regulation processes: implications for affect, relationships, and well-being. J Pers Soc Psychol. 2003;85(2):348–62. 12916575 10.1037/0022-3514.85.2.348

[73] Conway M, Csank PA, Holm SL, Blake CK. On assessing individual differences in rumination on sadness. J Pers Assess. 2000;75(3):404–25. 10.1207/S15327752JPA7503_04 11117154

[74] Hofmann SG, Kashdan TB. The Affective Style Questionnaire: Development and Psychometric Properties. J Psychopathol Behav Assess. 2010;32(2):255–63. 10.1007/s10862-009-9142-4 20495674 PMC2873215

[75] Gratz KL, Roemer L. Multidimensional assessment of emotion regulation and dysregulation: Development, factor structure, and initial validation of the difficulties in emotion regulation scale. Journal of psychopathology behavioral assessment. 2004;26(1):41–54.

[76] Salovey P, Mayer JD, Goldman SL, Turvey C, Palfai TP. Emotional attention, clarity, and repair: Exploring emotional intelligence using the Trait Meta-Mood Scale In: W PJ, editor. Emotion, disclosure, and health. Washington, DC: American Psychological Association; 1995 p. 125–54.

[77] Hayes SC, Strosahl K, Wilson KG, Bissett RT, Pistorello J, Toarmino D, et al Measuring experiential avoidance: A preliminary test of a working model. The psychological record. 2004;54(4):553–78.

[78] Nolen-Hoeksema S, Morrow J. A prospective study of depression and posttraumatic stress symptoms after a natural disaster: the 1989 Loma Prieta Earthquake. Journal of Personality & Social Psychology. 1991;61(1):115–21. 1890582 10.1037//0022-3514.61.1.115

[79] Meyer TJ, Miller ML, Metzger RL, Borkovec TD. Development and validation of the Penn State Worry Questionnaire. Behaviour Research & Therapy. 1990;28(6):487–95. 2076086 10.1016/0005-7967(90)90135-6

[80] Berking M, Znoj H. Entwicklung und Validierung eines Fragebogens zur standardisierten Selbsteinschätzung emotionaler Kompetenzen (SEK-27). Zeitschrift für Psychiatrie, Psychologie und Psychotherapie. 2008;56(2):141–53.

[81] Berking M, Orth U, Wupperman P, Meier LL, Caspar F. Prospective effects of emotion-regulation skills on emotional adjustment. J Couns Psychol. 2008;55(4):485–94. 10.1037/a0013589 22017555

[82] Berking M, Poppe C, Luhmann M, Wupperman P, Jaggi V, Seifritz E. Is the association between various emotion-regulation skills and mental health mediated by the ability to modify emotions? Results from two cross-sectional studies. J Behav Ther Exp Psychiatry. 2012;43(3):931–7. 10.1016/j.jbtep.2011.09.009 22406495

[83] Berking M, Meier C, Wupperman P. Enhancing emotion-regulation skills in police officers: results of a pilot controlled study. Behav Ther. 2010;41(3):329–39. 10.1016/j.beth.2009.08.001 20569782

[84] Berking M, Wupperman P, Reichardt A, Pejic T, Dippel A, Znoj H. Emotion-regulation skills as a treatment target in psychotherapy. Behav Res Ther. 2008;46(11):1230–7. 10.1016/j.brat.2008.08.005 18835479

[85] Brislin RW. Translation and content analysis of oral and written material In: Triandis HCaB, J. W., editor. Handbook of cross-cultural psychology: Methodology. Boston: Allyn and Bacon; 1980 p. 389–444.

[86] Geisinger KF. Testing and assessment in cross‐cultural psychology In: Graham JR, Naglieri JA, editors. Handbook of psychology. Vol. 10 New Jersey: NJ: John Wiley & Sons; 2003 p. 95–117.

[87] Van de Vijver F, Hambleton RK. Translating tests: Some practical guidelines. European psychologist. 1996;1(2):89–99.

[88] Bagby RM, Parker JD, Taylor GJ. The twenty-item Toronto Alexithymia Scale—I. Item selection and cross-validation of the factor structure. J Psychosom Res. 1994;38(1):23–32. 8126686 10.1016/0022-3999(94)90005-1

[89] Parker JDA, Taylor GJ, Bagby RM. The 20-Item Toronto Alexithymia Scale: III. Reliability and factorial validity in a community population. Journal of Psychosomatic Research. 2003;55(3):269–75. 12932802 10.1016/s0022-3999(02)00578-0

[90] Lovibond SH, Lovibond PF. Manual for the Depression Anxiety Stress Scales. 2nd ed Sydney: Psychology Foundation of Australia; 1995.

[91] Lovibond PF, Lovibond SH. The structure of negative emotional states: comparison of the Depression Anxiety Stress Scales (DASS) with the Beck Depression and Anxiety Inventories. Behav Res Ther. 1995;33(3):335–43. 7726811 10.1016/0005-7967(94)00075-u

[92] Derogatis LR, Melisaratos N. The Brief Symptom Inventory: an introductory report. Psychol Med. 1983;13(3):595–605. 6622612

[93] Lt Hu, Bentler PM. Cutoff criteria for fit indexes in covariance structure analysis: Conventional criteria versus new alternatives. Structural equation modeling: a multidisciplinary journal. 1999;6(1):1–55.

[94] MacCallum RC, Browne MW, Sugawara HM. Power analysis and determination of sample size for covariance structure modeling. Psychological methods. 1996;1(2):130.

[95] Marsh HW, Hau K-T, Wen Z. In Search of Golden Rules: Comment on Hypothesis-Testing Approaches to Setting Cutoff Values for Fit Indexes and Dangers in Overgeneralizing Hu and Bentler's (1999) Findings. Structural Equation Modeling: A Multidisciplinary Journal. 2004;11(3):320–41.

[96] Cheung GW, Rensvold RB. Evaluating goodness-of-fit indexes for testing measurement invariance. Structural Equation Modeling: A Multidisciplinary Journal. 2002;9(2):233–55.

[97] Hunt J, Eisenberg D. Mental health problems and help-seeking behavior among college students. J Adolesc Health. 2010;46(1):3–10. 10.1016/j.jadohealth.2009.08.008 20123251

[98] Hourigan SE, Goodman KL, Southam-Gerow MA. Discrepancies in parents' and children's reports of child emotion regulation. J Exp Child Psychol. 2011;110(2):198–212. 10.1016/j.jecp.2011.03.002 21458826

[99] Kramer U. Observer-rated coping associated with borderline personality disorder: an exploratory study. Clin Psychol Psychother. 2014;21(3):242–51. 10.1002/cpp.1832 23281000

[100] Diedrich A, Grant M, Hofmann SG, Hiller W, Berking M. Self-compassion as an emotion regulation strategy in major depressive disorder. Behav Res Ther. 2014;58:43–51. 10.1016/j.brat.2014.05.006 24929927

[101] Appelhans BM, Luecken LJ. Heart rate variability as an index of regulated emotional responding. Review of general psychology. 2006;10(3):229.

[102] LeMoult J, Joormann J. Depressive rumination alters cortisol decline in Major Depressive Disorder. Biol Psychol. 2014;100:50–5. 10.1016/j.biopsycho.2014.05.001 24835412 PMC4101056

[103] Giromini L, Velotti P, de Campora G, Bonalume L, Cesare Zavattini G. Cultural adaptation of the difficulties in emotion regulation scale: reliability and validity of an Italian version. J Clin Psychol. 2012;68(9):989–1007. 10.1002/jclp.21876 22653763

[104] Ebert DD, Christ O, Berking M. Entwicklung und Validierung eines Fragebogens zur emotionsspezifischen Selbsteinschätzung emotionaler Kompetenzen (SEK-ES). Diagnostica. 2013;59(1):17–32.

